# Randomized pilot trial of a synbiotic dietary supplement in chronic HIV-1 infection

**DOI:** 10.1186/1472-6882-12-84

**Published:** 2012-06-29

**Authors:** Marco Schunter, Hiutung Chu, Timothy L Hayes, Delandy McConnell, Sean S Crawford, Paul A Luciw, Stig Bengmark, David M Asmuth, Jennifer Brown, Charles L Bevins, Barbara L Shacklett, J William Critchfield

**Affiliations:** 1 Department of Medical Microbiology and Immunology, University of California, Davis, CA, USA; 2 Department of Pathology and Laboratory Medicine, Center for Comparative Medicine, University of California, Davis, California, USA; 3 Institute of Hepatology, University College of London, London, UK; 4 Division of Infectious Diseases, Department of Internal Medicine, University of California, Davis, Sacramento, California, USA

**Keywords:** Human immunodeficiency virus-1 (HIV-1), synbiotics, probiotics, prebiotics, microbial translocation, immune activation, highly active antiretroviral therapy (HAART), combined antiretroviral therapy (CART), complementary therapy.

## Abstract

**Background:**

Infection with HIV-1 results in marked immunologic insults and structural damage to the intestinal mucosa, including compromised barrier function. While the development of highly active antiretroviral therapy (HAART) has been a major advancement in the treatment of HIV-1 infection, the need for novel complementary interventions to help restore intestinal structural and functional integrity remains unmet. Known properties of pre-, pro-, and synbiotics suggest that they may be useful tools in achieving this goal.

**Methods:**

This was a 4-week parallel, placebo-controlled, randomized pilot trial in HIV-infected women on antiretroviral therapy. A synbiotic formulation (Synbiotic 2000®) containing 4 strains of probiotic bacteria (10^10^ each) plus 4 nondigestible, fermentable dietary fibers (2.5 g each) was provided each day, versus a fiber-only placebo formulation. The primary outcome was bacterial translocation. Secondary outcomes included the levels of supplemented bacteria in stool, the activation phenotype of peripheral T-cells and monocytes, and plasma levels of C-reactive protein and soluble CD14.

**Results:**

Microbial translocation, as measured by plasma bacterial 16S ribosomal DNA concentration, was not altered by synbiotic treatment. In contrast, the synbiotic formulation resulted in significantly elevated levels of supplemented probiotic bacterial strains in stool, including L. plantarum and P. pentosaceus, with the colonization of these two species being positively correlated with each other. T-cell activation phenotype of peripheral blood lymphocytes showed modest changes in response to synbiotic exposure, with HLA-DR expression slightly elevated on a minor population of CD4+ T-cells which lack expression of HLA-DR or PD-1. In addition, CD38 expression on CD8+ T-cells was slightly lower in the fiber-only group. Plasma levels of soluble CD14 and C-reactive protein were unaffected by synbiotic treatment in this study.

**Conclusions:**

Synbiotic treatment for 4 weeks can successfully augment the levels of probiotic species in the gut during chronic HIV-1 infection. Associated changes in microbial translocation appear to be absent, and markers of systemic immune activation appear largely unchanged. These findings may help inform future studies aimed at testing pre- and probiotic approaches to improve gut function and mucosal immunity in chronic HIV-1 infection.

**Trial registration:**

Clinical Trials.gov: NCT00688311

## Background

Infection with human immunodeficiency virus-1 (HIV-1) results in substantial dysfunction of the intestinal mucosa. During the past decade it has become clear that the intestinal mucosa is a site of vigorous virus replication beginning in the very early stages post-infection, and emerging from this storm of replication is a mucosa with a severely depleted CD4+ T-cell population as well as other notable gastrointestinal (GI) pathologies [1]. HIV-associated histopathology in the GI tract includes collagen deposition [2], degeneration of smooth muscle and enteric autonomic nerve fibers [3-5], and abnormal enterocyte morphologies such as villous blunting, vacuolization, and smooth endoplasmic reticulum changes [5,6]. In addition, HIV-1 and simian immunodeficiency virus (SIV) cause direct damage to intestinal epithelial cells [7,8], and gene expression studies of the GI mucosa have revealed HIV-associated upregulation of genes involved in inflammatory and apoptosis pathways [9]. In terms of the intestinal microbiota, HIV infection has been associated with depressed levels of beneficial bifidobacteria and lactobacilli, and elevated levels of opportunistic pathogens including *Pseudomonas aeruginosa* and *Candida albicans*[10]. In light of this broad set of impacts on the intestinal mucosa, it is not surprising that basic functional tasks of the gut, such as efficient nutrient absorption and the maintenance of intestinal barrier function, are significantly compromised [11-14].

Highly active antiretroviral therapy (HAART) regimens have proven to be very successful in terms of suppressing virus replication and promoting CD4+ T-cell reconstitution, but HAART does not provide a solution to the broad damage sustained by the GI mucosa. In fact, in the context of HIV treatments there are no therapies available to support or initiate the multiple repair processes which would be needed to restore full structural and functional integrity to the gut. In the absence of interventions to support such repair, there must certainly be health consequences to HIV-infected individuals. One such consequence is that HIV infection, even in the context of HAART, is associated with elevated microbial translocation from the gut into the circulation as evidenced by elevated plasma lipopolysaccharaide (LPS) and bacterial DNA levels [11,15]. In pathogenic SIV infection of rhesus macaques, a model system for HIV-1 infection in humans, circulating LPS is also elevated [11], and both bacteria and LPS can be detected in the intestinal lamina propria and mesenteric lymph nodes by immunostaining [16]. Importantly, both plasma LPS and plasma bacterial ribosomal 16S DNA levels in HIV patients are correlated with systemic immune activation markers such as CD8+ T-cells expressing both CD38 and HLA-DR [11,15]. Thus, a model has emerged where compromised gut barrier function results in elevated circulating LPS which fuels immune activation during chronic HIV-1. The importance of this is that immune activation, especially as measured by coexpression of CD38 and HLA-DR on CD8+ T-cells, is a better predictor of HIV disease progression than viral load or CD4+ T-cell loss [17]. Among HIV-infected patients, microbial translocation has also been found to be associated with sluggish CD4+ T-cell reconstitution in the context of HAART [15,18]. Thus, reduced gut barrier function in HIV infection has important health implications which point to a need to develop interventions to complement HAART.

Pre- and probiotics are prime candidates for therapies which may offer improvement in GI barrier function during chronic HIV-1 infection. A probiotic can be defined as ‘a live microbial food ingredient that is beneficial to health’ [19], and a prebiotic as ‘a nondigestible food ingredient that beneficially affects the host by selectively stimulating the growth and/or activity of one or a limited number of bacteria in the colon, and thus improves host health’ [20]. When pre- and probiotics are provided together they constitute a ‘synbiotic’ based on the potential to synergize. Broadly speaking, pre- and probiotics have been shown to exert numerous positive effects on GI function and immunity in human and animal studies. Examples of this, which are relevant to HIV-induced changes in the gut, include the ability of probiotics to: 1) maintain or enhance tight junction protein expression [21-24], 2) induce protective mucin production [25-27], 3) protect against enteroinvasive bacterial pathogens [26,28-30], and 4) decrease stress-induced bacterial translocation [31-34].

In the present pilot study, we evaluated Synbiotic 2000® (Medipharm, now owned by Synbiotic AB, Kustvägen 13, Sweden) in a double-blind, randomized, placebo-controlled parallel intervention. In previous human studies of trauma resulting from elective surgery or accidental injury, this formulation has been associated with significantly fewer bacterial infections and reduced need for antibiotic use [35-37], shorter duration of hospital stay [35], and reduced plasma levels of endotoxin and inflammatory cytokines [31,35]. The hypothesis for the present study was that ingestion of this synbiotic formulation would 1) enhance the levels of supplemented probiotic species in the gut, 2) improve gut barrier function as indicated by reduced microbial translocation, and 3) reduce systemic immune activation. We present results from assessments in a small cohort of HIV-1 infected women taking antiretroviral medications.

## Methods

### Ethics Approval and Study Design

This study was conducted with the approval of the Institutional Review Board of the University of California, Davis, USA, School of Medicine. Written informed consent was provided by all participants in the study.

This was a randomized, double-blind, placebo-controlled study. It was a parallel design, 4-week pilot study with an enrollment target of 38 subjects. The trial was conducted at the Center for AIDS Research, Education, and Services (CARES) in Sacramento, CA, between the dates of June 2008 and September 2009. There were no other trial sites.

Inclusion criteria were: 1) HIV seropositive, 2) adult female, 3) currently taking antiretroviral medications, and 4) blood CD4 count >200 cells/μl. These inclusion criteria were modified from an original form which called for a blood CD4 count of <500 cells/μl and for race limited to African-American and Caucasian. The change to the final form made the study available to a wider group of potential subjects without compromising the achievement of the scientific aims of the study. Exclusion criteria included: 1) Acquired immune deficiency syndrome (AIDS)-defining conditions, 2) current use of oral antibiotics, and 3) presence of inflammatory bowel disease or other known GI pathology.

The study interventions were 1) Synbiotic 2000® and 2) Fiber-only (the prebiotic components of Synbiotic 2000® alone). A single packet of Synbiotic 2000® consists of 4 strains of probiotic bacteria (10^10^ each) plus 4 nondigestible, fermentable dietary fibers (2.5 g each). The bacteria are *Pediococcus pentosaceus* 5–33:3, *Leuconostoc mesenteroides* 32*–*77:1, *Lactobacillus paracasei* subsp *paracasei* 19, and *Lactobacillus plantarum* 2362. The fibers include betaglucan, inulin, pectin, and resistant starch. The study intervention products were a gift of Medipharm, which had no other role in the study or its analysis.

The informed consent process was administered by the study coordinator. Subsequently, subjects made an appointment to begin the 4 week trial. At the initial visit, the study coordinator referred to a randomization schedule for assignment of participants to either the synbiotic or the prebiotic control interventions, and provided a supply of 30 packets to take home. Subjects were advised on how to store the packets and how to ingest the contents. Plastic tumblers with tight fitting lids were offered for dissolving the preparations in water or any beverage of the subjects’ choice, with the exception of alcoholic or hot drinks. A recordkeeping sheet was provided for participants to make an entry each day, noting their beverage of choice in ingesting the product. Subjects also indicated the frequency of probiotic-containing foods in their typical diet. Participants were reminded to notify the study coordinator in the event they needed to take oral antibiotics as this was one of the exclusion criteria. Sample collection at the initial visit included fresh stool and venous blood. Stool sampling was a modification to the original protocol, with 11 subjects from each group included in this analysis. This modification allowed for the determination of supplemented probiotic species in stool.

Approximately one week after the first visit, a follow-up phone call was made to the participants to check for any problems or concerns, and to offer encouragement in light of undesirable gas that can sometimes result from ingestion of these products. The second and final visit was on day 28, when subjects: 1) returned their recordkeeping sheet, 2) provided a fresh stool sample, and 3) supplied a venous blood sample.

### Randomization and blinding

Randomization to blocks was stratified based on subject-described race categories: 1) African-American, 2) Caucasian, and 3) other. These race categories were chosen based on the predominant makeup of the population of potential subjects visiting the CARES clinic. A randomization schedule was established by the primary investigator using a computer-based random number generator. The two interventions were supplied in daily dose packets with the same appearance other than the designation of cocktail A or cocktail B. Because the PI had knowledge of the identities of these cocktails, they were further coded to ‘X’ or ‘O’ and assembled into bags containing 30 packets of a given intervention (described above). The recoding scheme and bagging process was blind to all study personnel and was conducted by staff having no subsequent involvement in the study. During the course of the study, participants and the entire research staff remained blinded to treatment assignments, including the study coordinator, health care providers, study collaborators, student assistants, and the primary investigator.

### Outcomes and Sample Size

The primary outcome measure was the change from baseline in plasma bacterial DNA concentration. Secondary outcomes included changes in T-cell and monocyte phenotypes, blood CD4+ T-cell count, plasma soluble CD14 (sCD14), and plasma C-reactive protein (CRP). Shortly after the start of the study, stool collection was implemented in order to measure stool levels of supplemented probiotic bacteria as an additional secondary outcome. Sample size was based on power analysis using published data on plasma LPS levels in HIV infection [11] and employing 80% power, 5% level of significance, and an estimated treatment effect of 0.6 relative to the control. These parameters indicated a sample size of approximately 15 per group.

### Clinical Laboratory Assays

Blood CD4+ T-cell count was determined by the clinical laboratory at the University of California, Davis, USA, Medical Center. Plasma HIV loads were not measured as part of the study, but the most recent viral load relative to the start of the study was taken from the subjects’ medical record.

### Specimen Processing and DNA Isolation

Blood was collected into EDTA-containing tubes, centrifuged 10 min at 250 x g to obtain platelet-rich plasma, then processed using Ficoll-Paque^TM^ (GE Healthcare Bio-Sciences, Sweden) to derive peripheral blood mononuclear cells (PBMC). Plasma was stored at −80 °C and PBMC were cryopreserved in a liquid nitrogen freezer until later analyses. Aliquots of fresh stool weighing approximately 200 mg were taken using a 1 cc tuberculin syringe with the luer tip cut off, then placed in 2 ml microfuge tubes and stored at −80 °C until DNA isolation.

DNA was isolated from plasma using DNeasy® Blood & Tissue isolation kits (Qiagen, Maryland, USA) according to the manufacturer’s instructions. Each DNA preparation was made utilizing an input of 200 μl plasma that had been stored at −80 °C, and using a final elution volume of 200 μl at the end of the isolation protocol. DNA samples were stored at −80 °C until qPCR analysis. Stool DNA was isolated using the QiAamp® DNA Stool Mini Kit (Qiagen). The temperature of the lysing step was increased to 95 °C to improve the yield of DNA from gram positive bacteria in the specimens. DNA samples derived from stool were stored at −80 °C until qPCR analysis.

### Real-Time qPCR of Plasma Bacterial 16S DNA

Quantitative real-time PCR analysis of DNA samples from plasma was performed using a Roche LightCycler 2.0 coupled with FastStart DNA Master SYBR Green I detection. Reactions consisted of 10 μl input template (see above), 4 mM MgCl2, 0.5 μM of each primer, and buffered SYBR Green mix in a total reaction volume of 20 μl. Universal 16S bacterial primers were F909 5’-ACTCAAAKGAATTGACGG-3’ [38] and R1114 5’-GGGTTGCGCTCGTTRC-3’ [39]. The PCR conditions consisted of: Initial denaturation at 95 °C for 5 min then 45 cycles, each consisting of denaturation at 95 °C for 10 s, annealing at 50 °C for 5 s, and extension at 72 °C for 12 s. At the completion of the run melting curves were generated. Fluorescence was monitored at 530 nm and analyzed using LightCycler Software V 4.1 (Roche Diagnostics, Indiana, USA). A negative control lacking template, plus plasmid standards for absolute quantitation, were included with every set of reactions. In addition to absolute quantitation, samples were also evaluated for the melting temperature (Tm) of amplified products using LightCycler Software V 4.1.

### Real-Time qPCR of Synbiotic Bacterial Strains in Stool DNA

Using primers specific to the 4 bacterial species contained in the synbiotic supplement (see below), real-time qPCR was carried using a LightCycler 2.0 (Roche Diagnostics). Each reaction mixture (10 μl) contained 0.5 μM of each primer, 1.2 μl magnesium chloride, 1 μl LightCycler® FastStart DNA Master SYBR Green I and 1 μl (10 ng) of stool DNA. The PCR amplification program was 95 °C for 5 min, 45 cycles of 95 °C for 10 s, 58 °C for 5 s, 72 °C for 12 s, followed by 1 cycle of 65 °C for 15 s, and finally 40 °C for 30 s. An external standard curve for each of the four lactobacilli strains was developed for absolute quantitation. The plasmid stock solution of each strain, which contained approximately 10^11^ plasmid copies, was serially diluted in 0.02 μg/μl yeast RNA. 1 μl of each dilution was used in a total PCR volume of 10 μl to create the external standard curves. For quantitation of total bacterial 16S copies in stool samples, the standard curve for the *Pediococcus pentosaceus* plasmid was used.

### Design of primers for Real-Time qPCR of supplemented probiotic bacteria in stool

The four lactobacilli 16S rRNA gene sequences were compared with reference sequences from the Ribosomal Database Project release 9 which contained over 500,000 16S rRNA sequences. For each of the 4 lactobacilli strains a primer pair for the hypervariable region of the 16S rRNA gene sequence was designed using the software MacVector 9.5.2. To test the specificity of the primers the software Probe Match was used. The primers used for qPCR were: *Pediococcus pentosaceus* - sense: Ppent_117s (5’-ACACGAAGTGAGTGGCGAACG-3’), antisense: Ppent_271a (5’-CGGGTCCATCCAGAAGTGATA-3’), *Lactobacillus plantarum* - sense: Lplant_221s (5’-AGTTTGAAAGATGGCTTCGGCT-3’), antisense: Lplant_334a (5’-GATTACCCTCTCAGGTCGGCTA-3’), *Leuconostoc mesenteroides* - sense: Lmes_82s (5’-GCACCTTTTCAAGTGAGTGGCGAAC-3’), antisense: Lmes_162a (5’-TCTGTTTCCAAATGTTATCCCCAGC-3’), *Lactobacillus paracasei spp. paracasei* - sense: Lpara_150s (5’-AGTGGGGGATAACATTTGGAAACAG-3’), antisense: Lpara_296a (5’-CGCCTTGGTGAGCCATTACCTC-3’). To determine the total bacterial number in the stool the universal primers UniF334 (5’-ACTCCTACGGGAGGCAGCAGT-3’) and UniR514 (5’-ATTACCGCGGCTGCTGGC-3’) were used which detect about 80 – 85 % of the microflora in human stool.

### Construction of Plasmids for Absolute Quantitation in Real-Time qPCR

For plasmid standards applied to the measurement of stool levels of supplemented probiotic bacteria, genomic bacterial DNA was first isolated from Synbiotic 2000® by reconstituting one supplement packet in 500 ml water and then using the QIAamp DNA Stool Mini Kit (Qiagen) to obtain DNA. Next, 16S rRNA genes were amplified by conventional PCR using primers 27F (5’-AGAGTTTGATCCTGGCTCAG -3’) and 1492R (5’-GGTTACCTTGTTACGACTT-3’) which bind to conserved regions of the 16S rRNA gene. The settings for the PCR were 35 cycles at 94 °C for 30 seconds, 50 °C for 30 seconds and 72 °C for 2 min followed by a final elongation at 72 °C for 7 min. The PCR product was then purified (QIAquick PCR Purification Kit, Qiagen), and after enzymatic blunt end formation the resulting product was resolved with a preparative 1 %-agarose gel. Following extraction from the gel (QIAquick Gel Extraction Kit, Qiagen), the blunt end PCR insert was ligated into pBluescript II SK cloning vector and used to transform *Escherichia coli* DH5α cells (Invitrogen, Carlsbad, CA, USA). Plasmids were isolated with the QIAprep Spin Miniprep Kit (Qiagen), sequenced with a Big Dye Terminator V3.0 sequencing chemistry (Applied Biosystems, Carlsbad, CA) using vector-specific primers T3 and T7, and quantitated by absorbance at 260 nm.

Plasmid standards applied to the quantitation of plasma bacterial DNA were made in the same way as described immediately above, but by starting with a randomly selected stool DNA preparation from one of the study participants.

### Flow Cytometry

Cryopreserved PBMC were thawed and assessed for viability using trypan blue staining. Viability ranged from 87-98% and averaged 95%. With each batch of cells analyzed, a vial of internal control PBMC was included to assess run-to-run consistency of staining parameters. For each subject, PBMC from both the beginning and end of the study were always thawed, stained and analyzed in the same batch in order to provide consistency of analysis. Thawed cells (1–2 x 10^6^) were washed in PBS and surface stained for 20 min at room temperature in pre-titered fluorescently labeled antibodies plus Live/Dead® Fixable Aqua viability stain (Invitrogen,) in PBS in a total volume of 50 μl. Antibodies included CD3-Pac Blue (clone UCHT-1) and CD38-PE (Quantibrite^TM^, clone HB7) which were purchased from BD Biosciences (San Diego, CA, USA). CD4-APC (clone RPA-T4), CD8-APCAlexa750 (clone RPA-T8) HLA-DR-FITC (clone TU36), and CD14-Alexa700 (TuK4) were obtained from Invitrogen. PD-1-PerCP-eFlour710 (clone J105) was purchased from eBioscience (San Diego, CA, USA). Following surface staining, cells were washed in PBS/2% fetal calf serum and suspended in 1% formaldehyde prior to data acquisition. One hundred thousand lymphocyte events were acquired on a Becton-Dickinson LSRII flow cytometer equipped with 403 nm, 488 nm and 643 nm lasers.

Flow cytometry data were analyzed using FlowJo software (TreeStar, Ashland, OR, USA). Flourescence-minus-one stained cells were utilized to clarify gating where needed. Frequencies of CD4+ and CD8+ T-cells expressing CD38, HLA-DR, and PD-1 were established. Calculated frequencies of cell subsets expressing all possible combinations of these three markers were also derived using the Boolean gating feature in FlowJo. In addition to frequencies, the median fluorescence intensity (MFI) of CD38+ populations of T-cells was determined and converted to antibodies bound per cell using calibration beads (Quantibrite^TM^ PE Beads, BD Biosciences). Monocytes were also assessed for the MFI of CD14 and HLA-DR and for CD38 antibodies bound per cell.

### Plasma sCD14 and CRP

Plasma concentrations of sCD14 and CRP were measured in fresh-thawed plasma using Quantikine® ELISA kits purchased from R&D Systems (Minneapolis, MN, USA). Prior to assay, plasma was diluted 1:500 and 1:250 for sCD14 and CRP assays, respectively. Samples were assayed in duplicate and plates were read on a plate reader at 450 nm (Versamax, Molecular Devices, Sunnyvale, CA, USA). Curve fitting of standards and calculation of sample concentrations was done with SoftMax Pro software (Molecular Devices).

### Statistical methods

**Figure 1:**
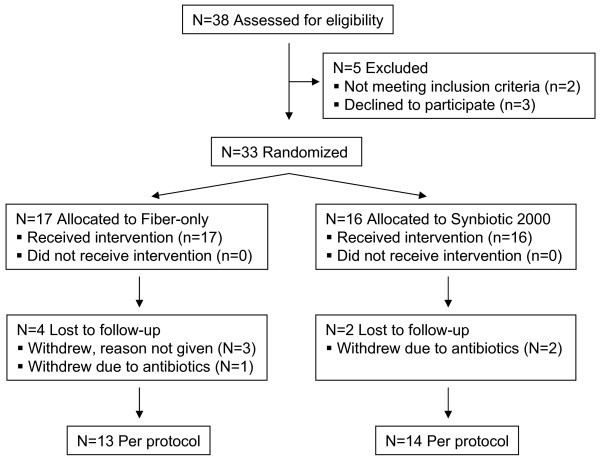
Participant Flow Diagram.

Statistical analyses were based on data derived from per protocol participants; i.e., data from subjects completing the study without any violations of protocol such as failure to complete the trial or taking oral antibiotics during the trial period (See Figure 1 for specific numbers). For both primary and secondary outcomes, the change from study baseline for a given parameter was calculated (i.e., Day 28 value minus baseline value). These change scores were compared between the two intervention groups using a Mann–Whitney test (2-tail in all cases). The significance of selected associations was evaluated using Spearman correlation (2-tail). Linear regression analysis was used to generate best-fit lines for visual (not statistical) purposes. For all statistical tests *p* < .05 was considered significant.

## Results

### Participant Flow Diagram and Characteristics of Study Subjects

The flow of participants in the study is shown in Figure 1. Thirty-eight subjects were assessed for eligibility, with 33 being randomized (1:1) to treatment interventions. The clinical characteristics of study participants assigned to each intervention are shown in Table 1. The two groups were similar in terms of the listed characteristics such as ethnicity, age, years on antiretroviral therapy, years of HIV seropositivity, baseline blood CD4+ T-cell count, plasma HIV load, and routine ingestion of cultured foods. Cultured foods included yogurt and other cultured dairy products, sauerkraut, kimchi, miso, tempeh, and others specified by the subject. All study participants were female and taking antiretroviral medication. Therefore, most subjects had plasma viral loads below the level of detection (<50 copies/ml), although 2–4 subjects per group did have detectable low level viremia. The geometric mean viral load per group for these few subjects is shown in Table 1.

**Table 1:** Characteristics of Study Participants at Baseline

**Characteristic**	**Fiber only Group**	**Synbiotic Group**
Gender, N (%)		
Female	13 (100)	14 (100)
Male	none	none
Race/Ethnicity, self described		
African American, N (%)	6 (46.2)	7 (42.9)
Caucasian, N (%)	5 (38.5)	6 (50.0)
Other, N (%)	2 (15.4)	1 (7.1)
Mean age, years (SD)	48.8 (6.1)	46.4 (8.0)
Years HIV seropositive, Mean (SD)	12.3 (6.8)	15.2 (8.6)
Years on antiretroviral therapy, Mean (SD)	7.0 (4.7)	8.7 (5.5)
Blood CD4 count, cells/μl, Mean (SD)	588 (309)	685 (249)
Viral load <50 copies/ml, N (%)	9 (69.2)	12 (85.7)
Viral load >50 copies/ml, N (Geometric Mean, SD)	4 (581, 6257)	2 (83, 1.4)
Cultured foods consumption. Mean servings/week (SD)	5.3 (4.1)	6.1 (5.4)

### Microbial translocation is quantitatively unaffected by synbiotic intervention

**Figure 2:**
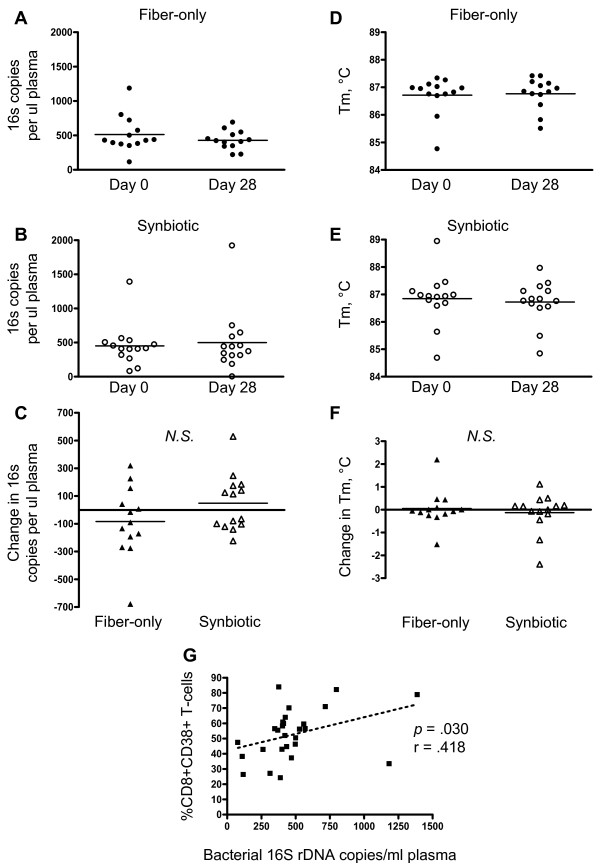
**Plasma bacterial 16S rDNA levels, Tm values of amplified products, and relationship to immune activation.** (**A**) Plasma 16S rDNA levels in the fiber-only group at baseline and day 28. (**B**) Plasma 16S rDNA levels in the synbiotic group at baseline and day 28. (**C**) Change scores for plasma 16S values calculated from paired values across the experimental period, presented for each group. (**D**) Fiber-only group Tm values at baseline and day 28. (**E**) Synbiotic group Tm values at baseline and day 28. (**F**) Tm change scores calculated from paired values across the experimental period for each intervention. (**G**) Spearman correlation of pretreatment plasma 16S rDNA concentration with pretreatment frequency of peripheral CD8 + CD38+ T-cells (all subjects at baseline). Horizontal bars denote the mean. N.S., not statistically significant (*p* > 0.05). r, Spearman r value.

To assess microbial translocation, we measured bacterial ribosomal DNA in plasma via real-time qPCR of a highly conserved sequence. Raw data on plasma concentration as well as calculated change scores are given in Figure 2A-C. An average of approximately 500 bacterial ribosomal 16S copies/μl plasma was measured at baseline and at Day 28 in each of the two intervention groups (Figure 2A and 2B). Evaluation of change scores indicated that the means of these changes were not statistically different between the two treatment groups (Figure 2C).

Melting profiles of amplified products can give an indirect indication of whether there are qualitative (rather than quantitative) differences in the products amplified from different time points. We therefore analyzed melting profiles to establish melting temperatures (Tm); in cases where there was more than a single peak, the primary peak was selected for analysis. Raw data and calculated change scores are shown in Figure 2D-F. The mean Tm was remarkably similar for both groups at both time points of the study, averaging close to 86.7 °C (Figure 2D and 2E). Change values calculated from these data are shown in Figure 1F and reveal that the average change in Tm for the two interventions was close to zero and was not statistically different (Figure 2F). Thus, using plasma 16S rDNA as an indicator of microbial translocation, we observed neither qualitative nor quantitative changes associated with synbiotic exposure relative to the fiber-only control.

Plasma bacterial 16S rDNA concentration in HIV patients has previously been shown to be correlated with coexpression of CD38 and HLA-DR on CD8+ T-cells [15]. Although our plasma 16S data did not indicate an effect of the synbiotic intervention on microbial translocation, it was of interest to explore whether in our cohort pretreatment levels of plasma 16S rDNA were related to pretreatment T-cell activation phenotypes as established by flow cytometry. Out of several possible activation phenotypes, we determined that the frequency of CD8 + CD38+ T-cells was most closely related to plasma bacterial 16S rDNA concentration, (p = .030, Figure 2G), supporting the concept of a relationship between microbial translocation and T-cell immune activation.

### Synbiotic treatment results in elevated fecal probiotic bacteria

**Figure 3:**
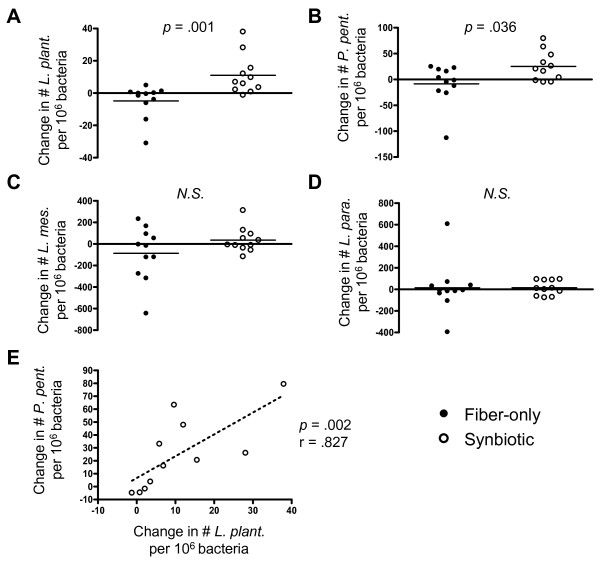
**Changes in fecal levels of supplemented probiotic bacteria.** DNA from fresh stool was amplified using primer sets specific to the supplemented bacteria and also using universal r16S primers to derive total fecal bacterial content. The number of r16S copies of each probiotic species in relation to one million total fecal r16S copies was calculated and the change in this value from baseline to day 28 shown here. (**A**) *Lactobacillus plantarum* 2362, (**B**) *Pediococcus pentosaceus* 5–33:3, (**C**) *Leuconostoc mesenteroides* 32–77:1, and (**D**) *Lactobacillus paracasei subsp paracasei* 19, (**E**) Spearman correlation between changes in *P. pent* and *L. plant* (Synbiotic group only)*.* Horizontal bars denote the mean. N.S., not statistically significant (*p* > 0.05). r, Spearman r value.

While the two interventions did not differ in terms of microbial translocation, it was possible that the synbiotic formulation altered the levels of supplemented probiotic bacteria in the gut. To this end, the presence of each probiotic strain was determined in fecal samples from 11 subjects in each group using real-time quantitative PCR (Figure 3). Of the four species in the synbiotic formulation, *L. plantarum* and *P. pentosaceus* showed significant increases (*p* = .001, *p* = .036, respectively) in stool concentration compared to the fiber-only intervention (Figure 3A, 3B), while the changes in *L. mesenteroides* and *L. paracasei* did not differ between the two treatments (Figure 3C, 3D). To explore whether the significant increases in *L. plantarum* and *P. pentosaceus* were related to each other, a Spearman correlation was calculated and revealed a significant positive correlation between the two species (Figure 3E, *p* = .002). Thus, the synbiotic intervention successfully modulated stool levels of probiotic bacteria which were also correlated with each other.

### T-cell and monocyte activation phenotypes remain largely unchanged with synbiotic intervention

**Figure 4:**
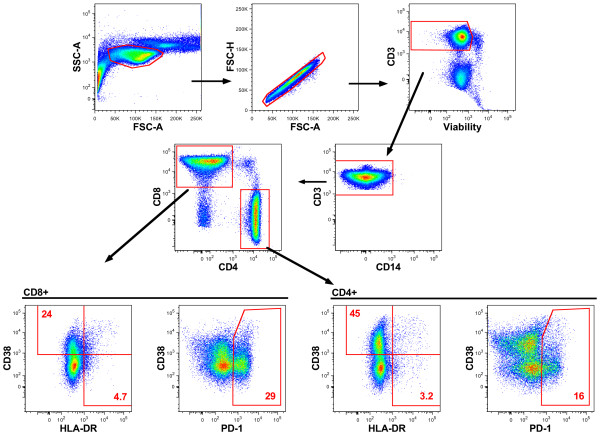
**Flow cytometry gating pathway for T-cell activation markers.** Initial gating was on lymphocytes as approximated by scatter, then doublets were eliminated. Next, live CD3+ cells were selected, CD14+ cells left behind, then CD4+ and CD8+ cells were gated. For cells in each of the CD4+ and CD8+ gates, individual gates for HLA-DR, CD38, and PD-1 were then established. The frequencies of these populations were analyzed as-is, and also after creation of Boolean combinations of these three groups.

We evaluated peripheral blood T-cell and monocyte phenotypic markers using flow cytometry, with Figure 4 showing the gating pathway utilized for the analysis of T-cells. For both CD4+ and CD8+ T-cells, gates were established for cells expressing the activation markers HLA-DR and CD38, and the T-cell exhaustion marker PD-1. The frequency of cells in these gates was analyzed in terms of the change from baseline for each subject in each intervention group. For a more detailed analysis, these three gates were also entered into Boolean combination analysis which generated 9 populations consisting of all possible combinations of expression (or not) of the 3 surface markers. A separate gating pathway using CD14 to identify monocytes was also employed (not shown). The monocyte population was assessed for the median fluorescence intensity (MFI) of CD14 and HLA-DR.

**Figure 5:**
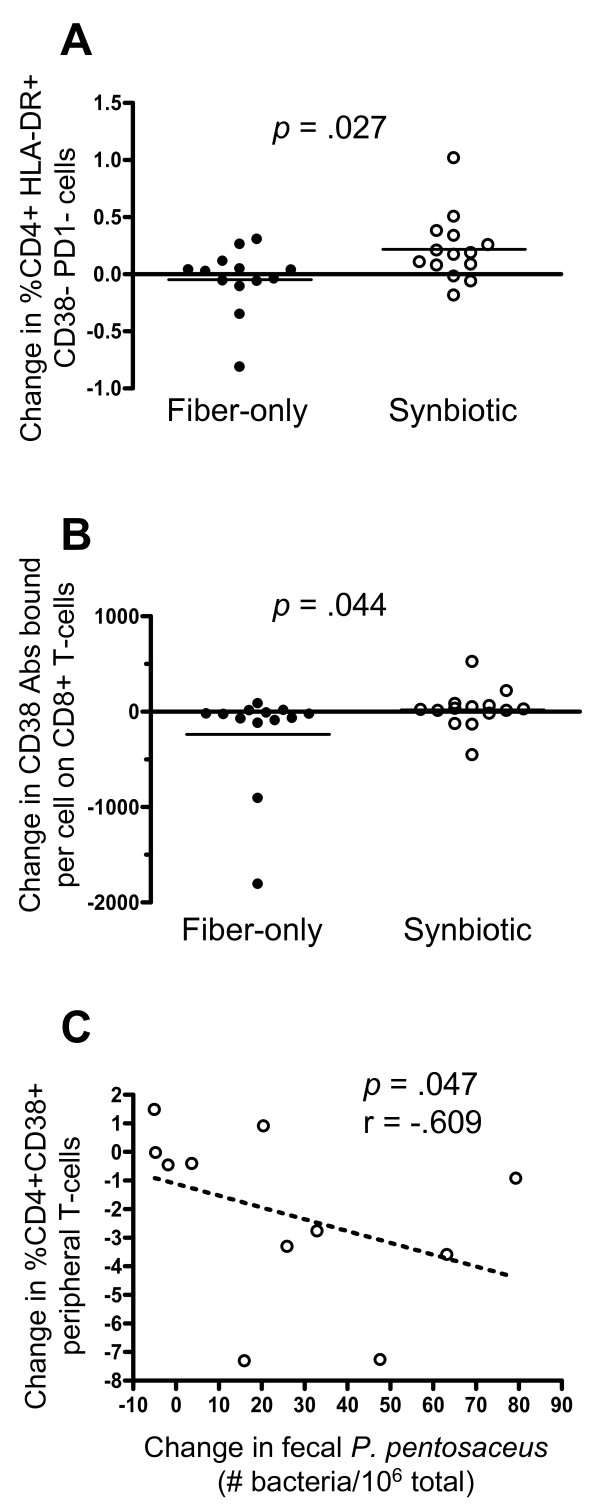
**Changes in CD4+ T-cell markers and association with level of supplemented bacterial in stool.** Surface expression of HLA-DR, CD38 and PD-1 were determined on peripheral blood lymphocytes using flow cytometry. These markers were quantified on CD4+ and CD8+ cells globally, and also using Boolean analysis (see Methods and Table 2) (**A**) Change in percent T-cells CD4 + CD38-HLADR + PD1-, (**B**) Change in number of CD38 antibodies bound per cell on CD8+ T-cells, (**C**) Within the Synbiotic group, correlation of the changes in CD38 expression on CD4+ T-cells with the change in fecal *P. pentosaceus*. Horizontal bars denote the mean. r, Spearman r value.

The expression patterns of CD38, HLA-DR, and PD-1 on CD4+ and CD8+ T-cells, the expression of CD14, CD38, and HLA-DR on monocytes, and blood CD4 count values are summarized in Table 2. The values are not change scores; rather, they are data corresponding to baseline and Day 28 for the two groups. Analysis of change scores yielded the *p* values in the far right column of Table 2. T-cell and monocyte activation phenotypes were remarkably stable during the study period for both of the interventions. One exception to this stability was a modest, but significant, synbiotic-associated increase in HLA-DR expression on a minor population of CD4+ T-cells that were negative for CD38 and PD-1 (*p* = .027, Table 2 and Figure 5A). A second finding was a difference in the change in CD38 antibodies bound per cell on CD8+ T-cells (*p* = .044, Figure 5B). The synbiotic group showed an average change of +19 antibodies bound per cell while the Fiber-only group had a change of −235.

**Table 2:** T-cell and Monocyte Phenotypes at Baseline and at Day 28

**Cell population**	**Parameter**	**Fiber-only**				**Synbiotic**				
		**Baseline**		**Day 28**		**Baseline**		**Day 28**		***p*value**
		**AVG**	**SD**	**AVG**	**SD**	**AVG**	**SD**	**AVG**	**SD**	
**CD4+ T-cells**	CD4+ T-cell count, cells/μl	627	293	619	337	683	259	697	296	*0.862*
	%CD38+	56.6	14.5	56.1	14.0	54.4	11.8	52.4	11.9	*0.216*
	CD38 Abs bound/cell	3737	980	3626	807	3353	1158	3276	1058	*0.790*
	%DR+	7.4	5.6	7.8	5.5	6.1	5.4	6.5	4.9	*0.827*
	%PD1+	23.5	10.5	24.5	9.2	25.2	12.2	26.0	11.7	*0.753*
	PD1, MFI	1315	282	1316	200	1212	152	1232	152	*0.610*
	%CD38 + DR+	3.8	4.2	4.1	3.8	2.7	1.9	2.8	1.5	*0.482*
	%CD38 + DR + PD1+	2.5	3.4	2.7	3.1	1.6	1.2	1.7	1.1	*0.452*
	%CD38 + DR + PD1-	1.3	1.1	1.5	1.1	1.1	0.9	1.1	0.6	*0.981*
	%CD38 + DR-PD1+	6.8	4.9	6.8	3.8	8.5	8.0	8.5	7.8	*0.716*
	%CD38 + DR-PD1-	46.0	16.1	45.1	14.4	43.2	13.6	41.1	12.7	*0.254*
	%CD38-DR + PD1+	1.7	1.1	1.8	1.3	1.5	1.5	1.6	1.3	*0.865*
	%CD38-DR + PD1-	1.9	1.3	1.8	1.3	1.9	2.6	2.1	2.8	***0.027***
	%CD38-DR-PD1+	12.5	6.1	13.1	6.5	13.6	4.9	14.3	5.0	*0.981*
**CD8+ T-cells**	%CD38+	54.3	17.8	54.4	16.7	51.1	15.1	49.3	15.2	*0.680*
	CD38, Abs bound/cell	2099	1365	1864	864	1803	661	1823	780	***0.044***
	%DR+	18.9	19.0	19.0	16.9	14.0	11.1	15.7	12.1	*0.254*
	%PD1+	28.5	12.5	29.4	13.3	20.8	9.4	23.7	11.4	*0.275*
	PD1, MFI	1423	211	1423	225	1280	163	1329	212	*0.235*
	%CD38 + DR+	13.4	15.5	13.5	13.5	8.2	6.0	9.1	7.3	*0.369*
	%CD38 + DR + PD1+	8.1	11.0	8.2	9.7	3.5	2.9	4.3	4.7	*0.512*
	%CD38 + DR + PD1-	5.4	5.4	5.3	4.8	4.6	4.2	4.9	4.4	*0.753*
	%CD38 + DR-PD1+	6.5	4.8	7.0	5.1	5.7	5.3	6.4	6.5	*0.680*
	%CD38 + DR-PD1-	34.4	13.9	33.9	13.2	37.3	11.2	33.8	10.6	*0.225*
	%CD38-DR + PD1+	2.7	3.0	2.8	2.8	2.2	2.5	2.5	2.9	*0.903*
	%CD38-DR + PD1-	2.7	3.5	2.7	3.1	3.6	6.3	4.1	7.0	*0.423*
	%CD38-DR-PD1+	11.2	7.2	11.4	7.6	9.3	4.8	10.5	4.9	*0.482*
**Monocytes**	CD14, MFI	13092	2255	12660	2176	13126	2323	13089	1855	*0.544*
	CD38, Abs bound/cell	11229	1868	11094	1658	11537	2764	11138	2458	*0.320*
	HLA-DR, MFI	10250	4641	9827	4393	10218	5988	9952	4079	*0.544*

*p* values refer to the comparison between Fiber-only and Synbiotic in the change from baseline. *p* values less than 0.05 are shown in bold font.

Given the small group sizes and the fact that fecal levels of supplemented bacteria varied among subjects in the synbiotic arm, we explored whether there could be correlations between fecal changes in *L. plantarum* or *P. pentosaceus* and immune cell activation phenotypes. To evaluate this, we selected parameters from Table 2 where the *p* value was less than 0.3 (there were 7 cases of this) and calculated Spearman correlations. Of these, a significant negative correlation emerged between the change in fecal *P. pentosaceus* and the change in % CD4 + CD38+ T-cells (Figure 5C) These data suggest a link between the gut colonization of synbiotic-derived *P. pentosaceus* and a drop in T-cell activation phenotype. In contrast, the significant elevation in HLA-DR expression on CD4+ T-cells negative for CD38 and PD-1, and the significant difference in CD38 antibodies bound per cell on CD8+ T-cells (Table 2 and Figure 5A, 5B) were not significantly correlated with changes in fecal *L. plantarum* or *P. pentosaceus* (data not shown).

### Plasma concentrations of sCD14 and CRP are unaltered by synbiotic treatment

**Figure 6:**
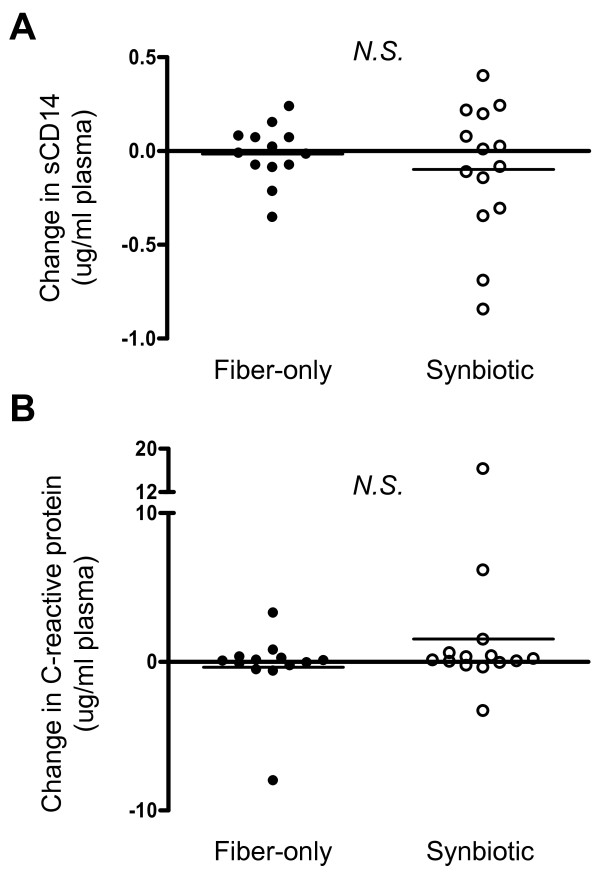
**Changes in soluble CD14 and C-reactive protein.** Plasma was assayed for sCD14 and C-reactive protein using ELISA. The change in concentration (μg/ml plasma) from baseline to day 28 was calculated for each subject and shown here according to intervention group. (**A**) Soluble CD14, (**B**) C-reactive protein. Horizontal bars denote the mean. N.S., not statistically significant (*p* > 0.05).

Circulating sCD14, released from monocytes in response to LPS exposure, as well as CRP produced by the liver, are both elevated in HIV infection and are each correlated with HIV disease progression [40,41]. These two factors were therefore measured in plasma using ELISA, with average plasma concentrations at baseline and day 28 being 1.9 and 1.9 μg/ml in the fiber-only group, and 2.1 and 2.0 μg/ml in the synbiotic group. Change values for each group are detailed in Figure 6, with average changes indicating no difference between the fiber-only and synbiotic groups. Levels of TNF-α and γ-IFN were also measured in plasma using Luminex® and showed no change in relation to treatment intervention (data not shown).

## Discussion

In this study we selected Synbiotic 2000® based on its documented activity in protecting hospitalized patients from infectious complications [35-37] and in reducing circulating inflammatory cytokines and LPS [31,35]. While the first part of our hypothesis, that probiotic species would be enhanced in the gut, was supported, the proposed drop in microbial translocation and immune activation was not observed. This could be interpreted as meaning that the chosen probiotic bacteria are intrinsically unable to exert the hypothesized downstream effects, that is, reduction of microbial translocation and immune activation. Conversely, our data raise the question of how much colonization, in terms of magnitude and duration, would be required to generate the hypothesized subsequent effects if they were to occur at all. To tackle this issue it seems necessary to design future studies of this type into two phases, the first being the testing of multiple formulations with variable dosing and time frames to establish optimal conditions for achieving substantial and reproducible enhancement in probiotic bacteria in the gut of HIV patients. A key issue to grapple with in this first phase lies in the fact that there is an enormous array of possible formulations to choose from. This is a reflection of the large diversity of probiotic species, the plethora of specific strains with unique properties, and the formulation of mixtures containing multiple probiotic species. With the addition of prebiotics as an additional variable, it becomes clear why the formulations in intervention studies vary so widely. The good news with this situation is that there is a rich, diverse palette available to the clinical scientist, while the negative aspect is that a formulation with bacteria lacking the desired properties may be chosen. As comprehensive studies on intestinal microbiota alterations in HIV infection become available, it may be possible to tailor interventions appropriately. These challenges aside, after establishing optimal formulations in phase one, the most promising strategies can then be tested in phase two to determine whether there are beneficial effects on gut and immune function.

The relevance of the modest changes in activation phenotypes (Table 2 and Figure 5) is not clear. The significant p-values were borderline and may have been influenced by the use of multiple comparisons. In addition, the difference in mean changes was small in both cases of statistical significance. For example, the phenotype consisting of CD4+ cells expressing HLA-DR but not CD38 or PD-1 is a very small population which changed a small amount (from 1.9 to 2.1 % of CD4+ T-cells). It would seem that if the modest change in this population in the synbiotic arm was a result of the synbiotic intervention then a correlation between this phenotype and stool levels of supplemented bacteria would be evident, which was not the case. Likewise, the other treatment-associated change, CD38 antibodies bound per CD8+ T-cell, did not correlate with stool probiotic bacteria either. Furthermore, the major activation phenotypes that we would have predicted to change in response to the synbiotic intervention (CD4+ and CD8+ T-cells double positive for HLA-DR and CD38) remained stable. Thus, the significance of the observed small shifts in T-cell subsets, and whether they actually resulted from experimental intervention remains in question.

Measuring microbial translocation requires accurate measurement of microbial constituents such as LPS or bacterial DNA in circulation. We chose to measure bacterial ribosomal 16S levels in order to take advantage of the reproducible and quantitative nature of real-time PCR. With this approach it is important to bear in mind that what is being measured is the presence of a highly conserved DNA sequence, 204 bp in our case, and not necessarily the presence of entire bacterial genomes or intact bacteria in circulation. Other studies have reported elevated plasma LPS and bacterial r16S concentration in HIV patients compared to HIV seronegatives [11,15]. While our study cohort was entirely HIV seropositive, we also measured r16S levels in the plasma of 10 volunteers at low-risk for HIV infection and found the average concentration to be reduced by 50% (data not shown). Related to bacterial translocation is the emerging surrogate marker sCD14 which is released from monocytes in response to LPS exposure, and in HIV patients is correlated with disease progression [41]. Our primary endpoint of microbial translocation was therefore assessed both directly via bacteria r16S and indirectly using the host factor sCD14. Data from these two methods support the conclusion that there was no change in microbial translocation. Finally, liver function may be relevant in studies of microbial translocation as the liver may remove incoming bacteria from the portal circulation to varying extents and the inclusion of markers of liver function therefore advisable. In this study, CRP, made by the liver as an acute phase protein, was also unaltered by the synbiotic.

Recent studies by other groups support the concept that pre- and probiotics may have positive impacts on CD4+ T-cell function in HIV infection [42]. For instance, Trois et. al. tested a probiotic formula containing *Bifidobacterium bifidum* and *Streptococcus thermophilus*, administered daily for 2 months to pediatric HIV patients on antiretroviral therapy, and observed a significant rise in blood CD4+ T-cell count (+118 cells/μl) compared to placebo (−42 cells/μl) [43]. A significant rise in blood CD4 T-cell count was also documented in separate studies of HIV infection carried out in Nigeria and Tanzania using a probiotic yogurt containing either *L. rhamnosus* Fiti [44] or *L. rhamnosus* GR-1 plus *L. reuteri* RC-14 [45]. Interestingly, a subsequent longer term 25 week study using *L. rhamnosus* GR-1 plus *L. reuteri* RC-14 did not reveal a CD4+ T-cell modulating effect, possibly due to the use of an encapsulated rather than a yogurt formulation [46]. Importantly, studies performed thus far have demonstrated safety in the administration of probiotics to HIV patients.

With regard to prebiotics, a pilot randomized 12 week trial of an oligosaccharide mixture in HAART-naïve HIV patients revealed reduced activation of peripheral CD4+ but not CD8+ T-cells (as measured by CD25 expression), but no change in blood CD4 T-cell count [47]. One explanation of these findings is that a drop in CD4+ T-cell activation may precede a subsequent, slower rise in CD4 count. In fact, in patients initiating HAART with CD4+ T-cell counts greater than 200 cells/μl, CD38 expression on peripheral CD4+ T-cells drops significantly prior to a rise in blood CD4 T-cell count [48]. Further, this pattern does not hold true for CD8+ T-cells, which lose CD38 expression more slowly after initiation of HAART. Given this pattern it seems likely that an additional incremental reconstitution of CD4+ T-cells in response to a pre- or probiotic intervention may be preceded by an initial drop in CD38 expression on CD4+ T-cells.

The present study had several limitations. First, because it was a pilot study the number of subjects enrolled was small and we focused on patients taking antiretroviral drugs. Had we been able to include patients with AIDS-defining characteristics, a group previously shown to have higher levels of immune activation and bacterial translocation than those taking antiretroviral drugs [11,15], the effects of the synbiotic may have been more clear. Another issue is that there was not a mechanism to reliably assess compliance, other than the subjects’ personal record keeping on ingesting the formulations which consistently indicated full compliance. Compliance seemed especially relevant given the unusual texture of the interventions and their tendency to produce intestinal gas, especially during the first week. A third limitation was the placebo being made up of the fiber component of the synbiotic. While this formulation did make it possible to implement double-blinded assignments, the fact that fiber alone can exert biological effects renders it a less than optimal placebo. Nevertheless, any differences between synbiotic and fiber-only can be taken as due to the probiotic component of the synbiotic. An additional limitation is that the measurement of circulating bacterial ribosomal 16S levels by qPCR does not yield information on the specific types of bacteria that are being translocated. The inclusion of sequencing to yield this information in future studies would be informative. Lastly, it was beyond the scope of this study to obtain intestinal mucosal biopsies. In response to pre-, pro- and synbiotics, some of the earliest and most important changes in immune cell phenotype and function may occur in the intestinal mucosa, and in this study we would have missed them.

## Conclusion

Treatment of HIV-1 infected subjects with a synbiotic for 4 weeks can successfully augment the levels of probiotic species in the gut, while associated changes in microbial translocation appear to be absent, and markers of systemic immune activation are largely unchanged. These data help provide a foundation for future studies aimed at optimizing synbiotic formulations and treatment schedules in HIV patients. With this information in hand, it will then be possible to determine whether synbiotics can improve gut barrier function and reduce chronic immune activation in this population. We remain optimistic that synbiotics may constitute an important complementary approach to antiretroviral therapy in the future.

## Competing interests

Author SB owns shares in Synbiotic AB, the company which provided the intervention products for the study. All other authors declare no competing interests. Synbiotic AB had no role in the design, implementation, analysis, or publication of the trial.

## Authors’ contributions

MS designed and performed stool PCR experiments. HC designed and contributed to plasma PCR experiments. TH processed specimens and contributed to flow cytometry panel design. DM processed specimens and acted as clinical coordinator. SC participated in sample processing and data collection. PL contributed to measurement of circulating inflammatory markers. SB participated in conceiving and designing the study. DA contributed to study design and subject recruitment. JB coordinated subject recruitment. CB helped design, coordinate and interpret PCR studies. BS contributed to study conception and design, and flow cytometry panel design. JC conceived and designed the study, obtained IRB approval and secured pilot funding, contributed to PCR studies, performed flow cytometry experiments and data analysis, and wrote the manuscript. All authors read and approved the final manuscript.

## Pre-publication history

The pre-publication history for this paper can be accessed here:

http://www.biomedcentral.com/1472-6882/12/84/prepub
